# The effect of human PBMCs immobilization on their Аβ42 aggregates-dependent proinflammatory state on a cellular model of Alzheimer’s disease

**DOI:** 10.3389/fnins.2024.1325287

**Published:** 2024-02-09

**Authors:** Kateryna Kot, Yurii Kot, Rustam Kurbanov, Hanna Andriiash, Olena Tigunova, Yaroslav Blume, Sergiy Shulga

**Affiliations:** ^1^Biochemistry Department, V. N. Karazin Kharkiv National University of Ministry of Education and Science of Ukraine, Kharkiv, Ukraine; ^2^Department of Genomics and Molecular Biotechnology, Institute of Food Biotechnology and Genomics National Academy of Science of Ukraine, Kyiv, Ukraine

**Keywords:** Alzheimer’s disease, Аβ42 aggregates, hPBMCs, β1 integrins, cytokines

## Abstract

The leading pathological mechanisms of Alzheimer’s disease are amyloidosis and inflammation. The presented work was aimed to study the effect of human peripheral blood mononuclear cells (hPBMcs) cells-matrix adhesion on their pro-inflammatory state *in vitro*. Although direct interaction of Аβ42 to PBMC is not a cellular model of Alzheimer’s disease, PBMCs may serve as test cells to detect Аβ42-dependent molecular effects in monitoring disease progression. Peripheral blood mononuclear cells (PBMCs) are used to assess changes in cytokines released in response to diseases or Alzheimer’s disease-specific cytotoxic molecules such as Aβ42. The effect of recombinant amyloid β-peptide rАβ42 on the concentration of endogenous amyloid β-peptide Aβ40 and pro-inflammatory cytokines TNFα and IL-1β in human peripheral blood mononuclear cells that were cultured in suspension and immobilized in alginate microcarriers for 24 h were investigated. The localization and accumulation of Aβ40 and rAβ42 peptides in cells, as well as quantitative determination of the concentration of Aβ40 peptide, TNFα and IL-1β cytokines, was performed by intravital fluorescence imaging. The results were qualitatively similar for both cell models. It was determined that the content of TNFα and Aβ40 in the absence of rAβ42 in the incubation medium did not change for 24 h after incubation, and the content of IL-1β was lower compared to the cells that were not incubated. Incubation of cells *in vitro* with exogenous rAβ42 led to an increase in the intracellular content of TNFα and Aβ40, and no accumulation of IL-1β in cells was observed. The accumulation of Aβ40 in the cytoplasm was accompanied by the aggregation of rAβ42 on the outer surface of the cell plasma membrane. It was shown that the basic levels of indicators and the intensity of the response of immobilized cells to an exogenous stimulus were significantly greater than those of cells in suspension. To explore whether non-neuronal cells effects in alginate microcarriers were cell-matrix adhesion mediated, we tested the effect of blocking β1 integrins on proamyloidogenic and proinflammation cellular state. Immobilization within alginate hydrogels after incubation with the β1 integrins blocking antibodies showed a remarkable inhibition of TNFα and Aβ40 accumulation in rAβ42-treated cells. It can be concluded that activation of signal transduction and synthesizing activity of a portion of mononuclear cells of human peripheral blood is possible (can significantly increase) in the presence of cell-matrix adhesion.

## 1 Introduction

It is generally known, that abnormal accumulation of amyloid β-peptides (Aβ) in brain cells is associated with pathological processes in Alzheimer’s disease. Among all types of amyloid β-peptide isoforms, the Aβ40 and Aβ42 isoforms are considered the most important in the development of Alzheimer’s disease (Fontana et al., 2020). Although these isoforms differ by only two amino acid residues, the results of *in vivo* and *in vitro* studies indicate that they differ significantly in physicochemical properties, metabolism, and the degree of gene-and cytotoxicity (Qiu et al., 2015; Welty et al., 2022; Zhang et al., 2023). Aβ40 is more common and Aβ42 is more prone to aggregation and is considered more pathogenic due to its increased hydrophobicity. The results of Meisl et al. show that the significant differences in the observed aggregation behavior of the two proteins are rather due to a shift of more than one order of magnitude in primary nucleation versus secondary fibril-catalyzed nucleation processes (Sandebring et al., 2013; Meisl et al., 2014). Previous research showed that the synthesis of precursor proteins of amyloid β-peptides in peripheral blood mononuclear cells is enhanced under conditions of cellular activation by various ligands, including exogenous Aβ42. In particular, rAβ42 stimulation has been shown to induce the production of proinflammatory cytokines IL-1β, IL-6, IFN-γ, and TNF-α, as well as anti-inflammatory cytokines IL-10 and IL-1Ra (Ledoux et al., 1993; Pellicanò et al., 2010). One of the most essential factors in the activation of peripheral blood mononuclear cells is also cell–cell and cell-matrix adhesion (Vanoni et al., 2022; Haydinger et al., 2023). Cell models undoubtedly allow a deeper understanding of the destructive effect of amyloid β-peptides on cells, and contribute to the development of innovative methods of treating Alzheimer’s disease. Cultivation in suspension of peripheral blood mononuclear cells of healthy people and patients with Alzheimer’s disease is a classic cell model for studying the processes of neuroinflammation, amyloidogenesis, and the influence of exogenous Aβ42 (Cetin et al., 2022). However, the suspension model does not include the factor of cell activation by adhesion, which always occurs *in vivo* and is a striking difference between *in vivo* and *in vitro* systems. In immune cells, there are many essential adhesion molecules that determine the formation of their subpopulations and functional specificity (Tan, 2012). However, in order to implement their signaling action, cells in suspension are limited by such factors as the available surface area of the plasma membrane, the number and density of adhesion sites, and the ability to deform cell membranes. Failure to consider the cell adhesion factor and the use of suspension culture may be the cause of false conclusions in the study of exogenous stimuli on cells obtained from healthy donors and donors with Alzheimer’s disease. For example, the intensity of production of free oxygen species by monocytes is controlled by integrin adhesion receptors (Garnotel et al., 2000), and peripheral blood mononuclear cells derived from Alzheimer’s disease patients show elevated baseline levels of secreted cytokines but resist stimulation with β-amyloid peptide when cultivated in suspension after isolation from peripheral blood (Rocha et al., 2012). Currently, there are various methodical approaches for introducing the adhesion and migration factor into the culture of peripheral blood mononuclear cells when studying the influence of exogenous factors. The classic approach is cultivation using Transwell technology. Studies using Transwell inserts are very popular due to their simplicity (Schimpel et al., 2023). However, this approach has several significant methodological limitations. Firstly, single cells cannot be visualized by fluorescence confocal microscopy on the surface and in the thickness of an opaque polycarbonate porous membrane, which does not allow high-resolution analysis of the localization of an exogenous factor or cellular metabolites in the cell. Secondly, cultivation systems based on Transwell technology do not allow the collection of cells from the thickness of the carrier without damaging them or effectively lyse them for further studies. Thirdly, such a cultivation process is static, it does not occur in the flow of the nutrient medium and leads to the formation of gradients of influencing factors and metabolites, which complicates the standardization of studies (Vanoni et al., 2022). These shortcomings can be solved by using the GEM (Global Eukaryotic Microcarrier) technology, which allows not only immobilization and cultivation of cells on the surface of transparent spherical microcarriers made of alginate but also constant maintenance of cells in suspension, which brings the cultivation conditions markedly closer to *in vivo* conditions (Tavassoli et al., 2018; Xiang et al., 2023). In view of the above, the aim of the presented work was to compare the intensity of the response of cells in suspension and immobilized cells as part of GEM microcarriers to the exogenous Aβ42 stimulus.

## 2 Materials and methods

### 2.1 Primary cells source and storage

The experiments were performed with mononuclear cells of human peripheral blood (Abcell-bio, Ref. 1,006-50 M). According to the description of the distributor human peripheral blood mononuclear cells (hPBMCs) were isolated from blood an adult single healthy donor using the Ficoll–Hypaque method and frozen in CryoStor^®^ CS10 medium (STEMCELL Technologies). The distributor has performed multiple quality controls, virologic (HIV1/2, HBV, HCV) and mycoplasma detection tests, certifying their virologic conformity and viability. In the laboratory, cells were stored in Biorack 3,000 cryogenic storage (Statebourne Cryogenics, United Kingdom). Immediately before culturing, cells were thawed and washed from cryoprotectant according to the protocol (BD Biosciences, 2015) and their number, viability and size distribution were determined. The status of mycoplasma contamination of cultured cells was determined by MycoAlert Mycoplasma Detection Kit (Lonza Bioscience) and Sirius L luminometer (Berthold Technologies). All tests were performed in triplicate.

### 2.2 Cells quantity, size, and viability assay

Determination of the number, viability and size heterogeneity of cells in the primary cell suspension was carried out by the method of measuring impedance in the flow, using an automatic cell counter Scepter 2.0 (Millipore) and Scepter Software Pro 2.1 (Millipore). The primary cell suspension was examined for the total number of cells (1.5 × 10^6^/ml), cell viability (92.0%) and the degree of heterogeneity in size (4–18 μm). The obtained data on the distribution of cells by size are consistent with the data presented in other publications (Mardi et al., 2010; Lengefeld et al., 2021). Intravital cell morphology was assessed by confocal microscopy after staining nuclear DNA with DAPI dye (Abcam, ab228549, λEx – 405 nm, λEm = 461 nm). The hPBMCs studied included all mononuclear blood cells, such as lymphocytes, monocytes, and stem cells.

### 2.3 Suspension cells culture

All manipulations with cells were carried out in aseptic conditions. hPBMCs were resuspended in RPMI-1640 nutrient medium (Gibco, cat. no. 11875093) containing 10.0% FBS (Gibco, cat. no. A3840302), 1.0% glutamine, 1.0% pyruvate and placed in the wells of a 24-well plate (Cellvis, cat. no. P24-1.5H-N) to have a final concentration of 1.5 × 10^6^ cells/ml. hPBMCs were cultured for 24 h (37.0 ± 0.1°C, 5.0 ± 0.1% CO_2_, 99.5 ± 0.1% RH, orbital mixing at 7 revolutions/h) in the Galaxy 14S CO_2_ incubator (Eppendorf).

### 2.4 Immobilized cells culture

Cultivation of hPBMCs in an immobilized state was carried out using the GEM - Global Eukaryotic Microcarrier technology, according to which cells are immobilized on the surface of spherical alginate microcarriers. Alginate microcarriers were prepared using the GEM Microcarrier Packs kit (Global Cell Solutions, cat. no. GEM-4133) and following the manufacturer’s instructions. Because hPBMCs have a high degree of adhesion to collagen and fibronectin (Chen et al., 2017), the alginate solution contained 1.0% collagen type 1 (Merck, cat. no. CC050), 0.5% RGD (Arg-Gly-Asp) peptide (Abcam, cat. no. Ab142698), and 1.0% fibronectin (Merck, cat. no FC010). Microcarriers were stored at 4.0°C in PBS until use. Before immobilization of hPBMCs, alginate microcarriers were washed three times with RPMI-1640 medium (Gibco, cat. no. 11875093), and preheated to 37.0°С. Cells were added to the suspension of alginate carriers at the rate of 0.5 × 10^6^ cells/ml of suspension. After 24 h, the cells adhered to the surface of the microcarriers. Non-attached cells were removed when the nutrient medium was replaced. Fifteen microliter of the microcarrier suspension with immobilized hPBMCs was placed in a LeviTube vial (OMNI Life Sciences, cat. no. #2800005) and cultured for 24 h (37.0 ± 0.1°C, 5.0 ± 0.1% CO_2_, 99.5 ± 0.1% RH, mixing 5 revolutions/h) in the CERO BioLevitator bioreactor (Omni Life Science GmbH & Co). The conditions for the production of media, immobilization and cultivation of hPBMCs were in accordance with the current practice of dynamic cultivation of this type of cells (Ngo et al., 2022).

### 2.5 Cells incubation with Аβ42

A sterile suspension of recombinant human β-amyloid peptide rАβ42 (Abcam, cat. no. ab120301, CAS 107761-42-2) in endotoxin-tested HyClone Water (Cytiva, cat. no. SH30529.01) was added to the nutrient medium to a final concentration of 15 nM.

Aβ in the metastable zone of supersaturation (10–20 nM) does not spontaneously initiate aggregation, but can aggregate in the presence of pre-formed aggregates after additional trigger factors such as Aβ to cells contacts (Portugal and Guo, 2023). Previously the aggregation status of rАβ42 was verified. Fluorescence microscopic examination of the rAβ42 aggregation process in cell-free medium by fluorescent dye ThT for specific imaging of the aggregation detection was performed. There was no spontaneous rAβ42 aggregation in cell-free medium when Aβ concentration was 15 nM, rAβ42 aggregation was observed at rAβ42 concentrations higher than 300 nM. This is confirmed by literature data (Hellstrand et al., 2010). Nevertheless, immediately prior to addition, the rAβ42 suspension was sonicated with cooling in a Biocision CoolRack (4.0°C) for 3 s on a Fisher Model 100 sonicator (10 kHz) to disperse peptide aggregates that may have formed.

### 2.6 Beta-1 integrins blocking

To explore whether hPBMCs immobilization effects in alginate microcarriers were cell to matrix adhesion mediated, β1 integrins were blocked with an anti-β1 integrin antibody and pro-amyloidogenic and pro-inflammation cellular parameters were assessed. The hPBMCs were incubated with anti-β1 integrin antibody (Abcam, cat. no. ab24693, 1:200) in serum-free RPMI-1640 nutrient medium (Gibco, cat. no. 11875093) for 30 min at 37°C. Cells were then encapsulated in alginate microcarriers as described above and at hours 24 the pro-amyloidogenic and pro-inflammation parameters assays were performed.

### 2.7 TNFα, IL1β, and Аβ40 assay

Determination of the content of pro-inflammatory cytokines TNFα, IL1β, and endogenous β-amyloid peptide Aβ40 in the lysate of human peripheral blood mononuclear cells was performed after 1 and 24 h of incubation with rAβ42 by immunoenzymatic method with spectrophotometric detection on a FL600 microplate multimodal reader (BioTek) using test kits “Human TNF alpha ELISA Kit” (Abcam, cat. no. ab181421), “Human IL-1 beta ELISA Kit” (Abcam, cat. no. ab214025) and “Amyloid beta 40 Human ELISA Kit” (Thermo Scientific, cat. No. #KHB3481) respectively. NP-40 buffer (Thermo Scientific, cat. no. J60766.AP) was used for cell lysis. Determination of total protein in cell lysate was carried out by the direct UV spectrophotometric method on a scanning spectrophotometer Ultrospec 3,100 pro (Biochrom). The content of cytokines and endogenous Aβ40 was calculated in ng/g of total protein.

### 2.8 Imaging of rАβ40 and Аβ42 in live cells

In addition to the quantitative determination of the content of endogenous β-amyloid peptide Aβ40 after exposure to exogenous rAβ42, intravital visualization of the accumulation of Aβ40 and rAβ42 in cells was performed using fluorescent NIR probes specific to these peptides CRANAD-2 (Abcam, cat. no. ab141775) and MCAAD-3 (Abcam, cat. no. ab216983) respectively. Aliquots of cell suspensions and alginate media were centrifuged (200 g, 10 min, room temperature) in a Durafuge 300 centrifuge (Thermo Scientific). Cell-free culture medium was disposed of in accordance with biosafety regulations (Fleming and Hunt, 2000). Cells were resuspended in Invitrogen Live Cell Imaging Solution (Life Technologies, cat. no. A14291DJ) prewarmed to 37.0°C. Visualization was performed on a laser scanning confocal microscope FV10i-LIV (Olympus) equipped with a 60/1.2 NA water immersion objective and a system of intravital cell incubation (37.0 ± 0.1°C, 5.0 ± 0.1% CO_2_, 99.5 ± 0.1% RH). Cells cultured in suspension were visualized in single-well PTFE slides (Thermo Scientific, cat. no. X2XER203B#), covered with coverslips (Ibidi, cat. no. 10812). Visualization of cells in GEM carriers was performed in confocal dishes (Ibidi, cat. no. 81158). The analysis of the localization and content of Aβ40 and rAβ42 in cells was carried out according to the ratiometric index R - the ratio of rAβ42-specific fluorescence intensity (If 685 nm) to Aβ40-specific fluorescence intensity (If 715 nm).

Confocal images were acquired with a scanning mode format of 1,024 × 1,024 pixels. The pinhole aperture was 1 Airy unit. Z-reconstruction of serial single optical sections was performed with a scanning mode of 1,024 × 1,024 pixels with an electronic zoom at 2.0 and a Z stack of 1.0 μm/slice. Confocal images shown are representative images of six fields of view in different regions of single-well PTFE slides (for suspended cells) or confocal glass bottom dish (for GEM carriers). For triplicate samples, 50 cells per field of view were scored. Post-rendering of the obtained images of optical sections, measurements of fluorescence intensities and ratiometric analysis were performed using Olympus cellSence software (Olympus licensed).

### 2.9 Statistical analysis

Statistical differences were determined by one-way analysis of variance (ANOVA) followed by Tukey test for multiple comparisons. All analyses were performed using Statistica 7.0 software (StatSoft Inc.) for Windows. Data were presented as mean ± standard error of the mean (SEM). Differences were considered significant at *p* < 0.05.

## 3 Results

**Figure 1:**
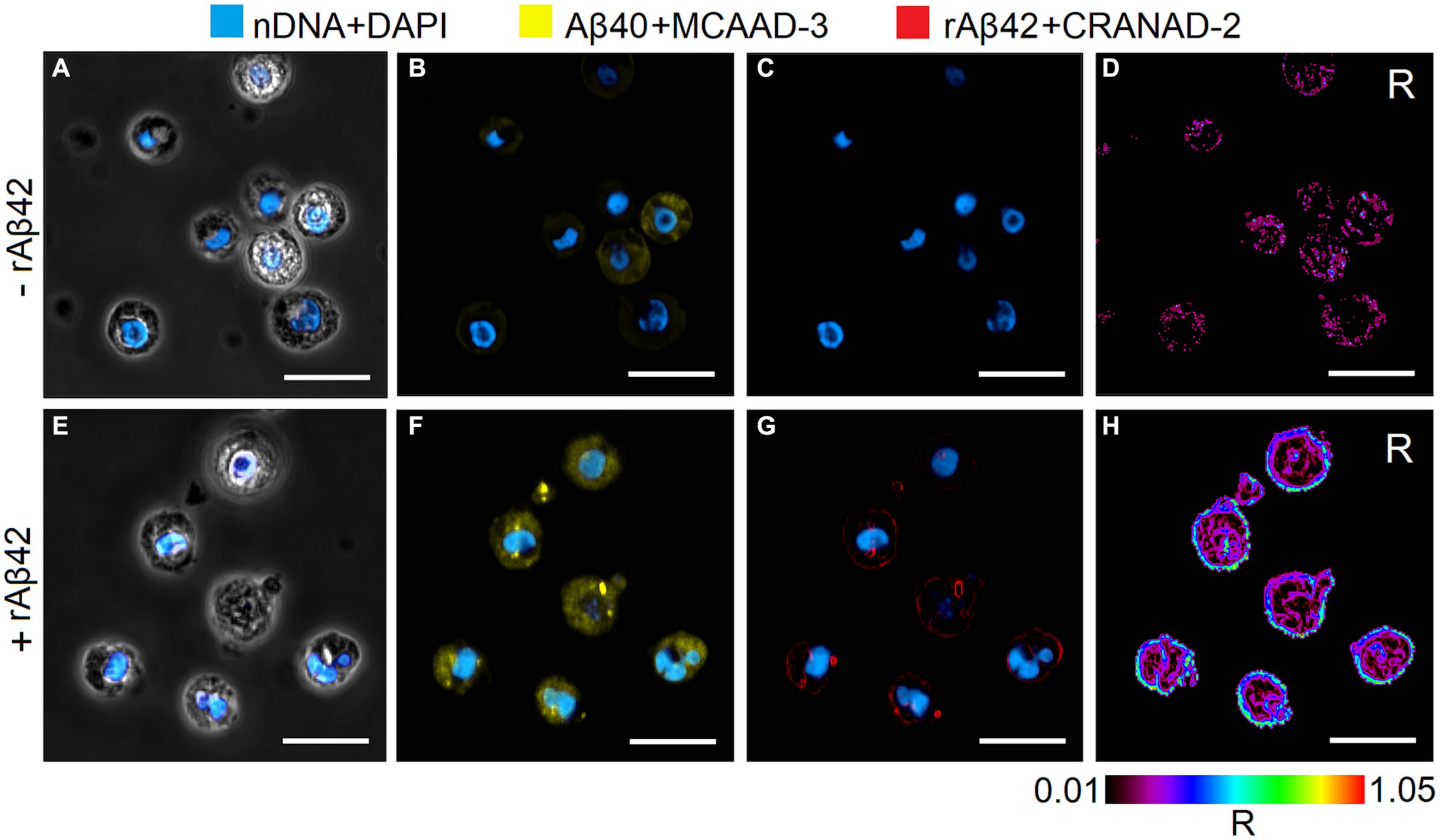
The representative imaging of suspended hPBMCs (*n* = 50, 1n = single analyzed cell) without **(A–D)** and after 24 h incubation **(E–H)** with rАβ42 (15 nM) by laser scanning confocal microscopy. Blue – nuclear DNA staining by DAPI dye; yellow – Аβ40 staining by MCAAD-3; red – rАβ42 staining by CRANAD-2. R – ratiometric imaging (rАβ42/Аβ40). Scale bar = 10 μm.

**Figure 2:**
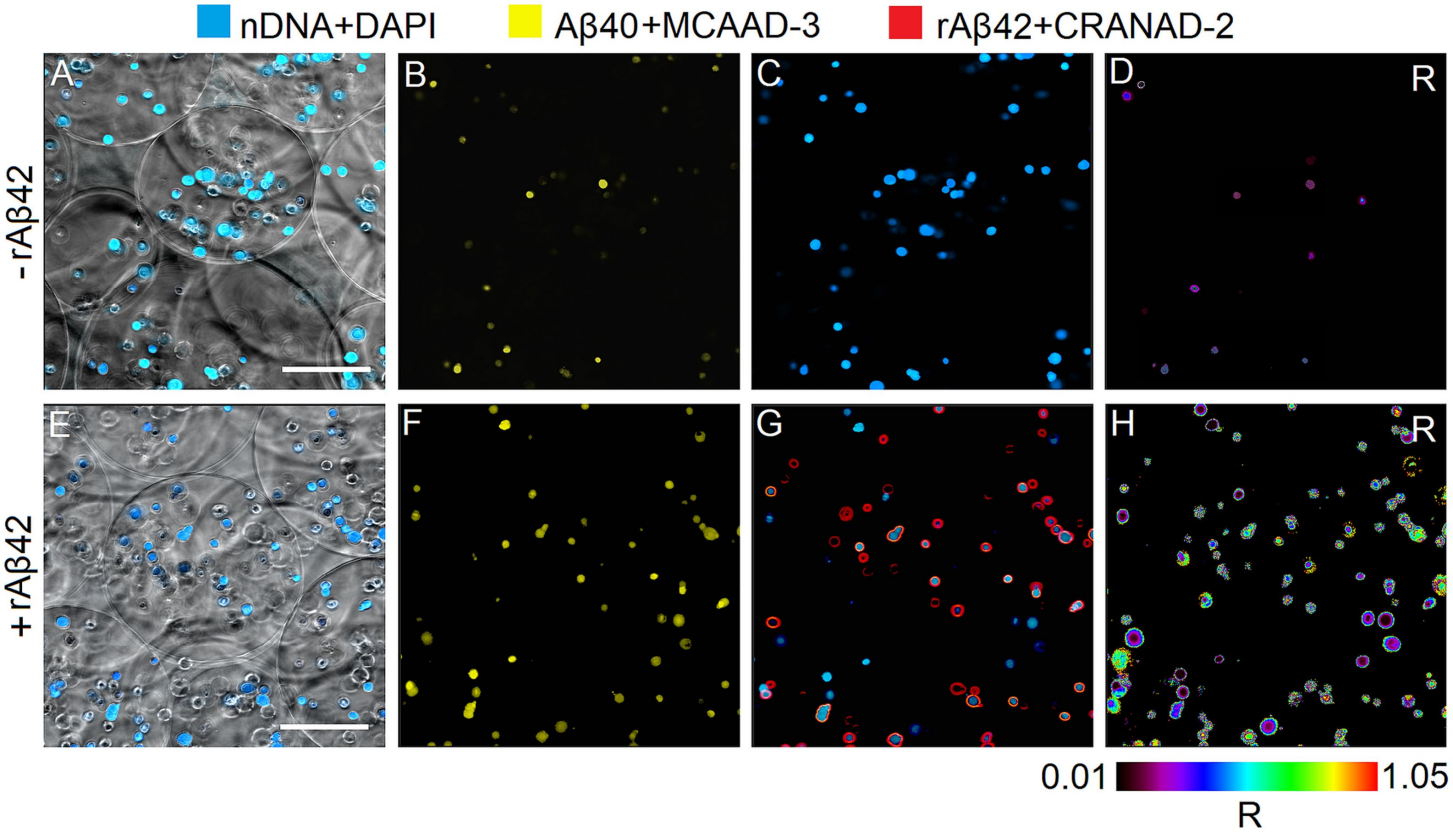
The representative imaging of immobilized hPBMCs (*n* = 50, 1n = single analyzed cell) without **(A–D)** and after 24 h incubation **(E–H)** with rАβ42 (15 nM) by laser scanning confocal microscopy. Blue – nuclear DNA staining by DAPI dye; yellow – Аβ40 staining by MCAAD-3; red – rАβ42 staining by CRANAD-2. R – ratiometric imaging (rАβ42/Аβ40). Scale bar = 150 μm.

**Figure 3:**
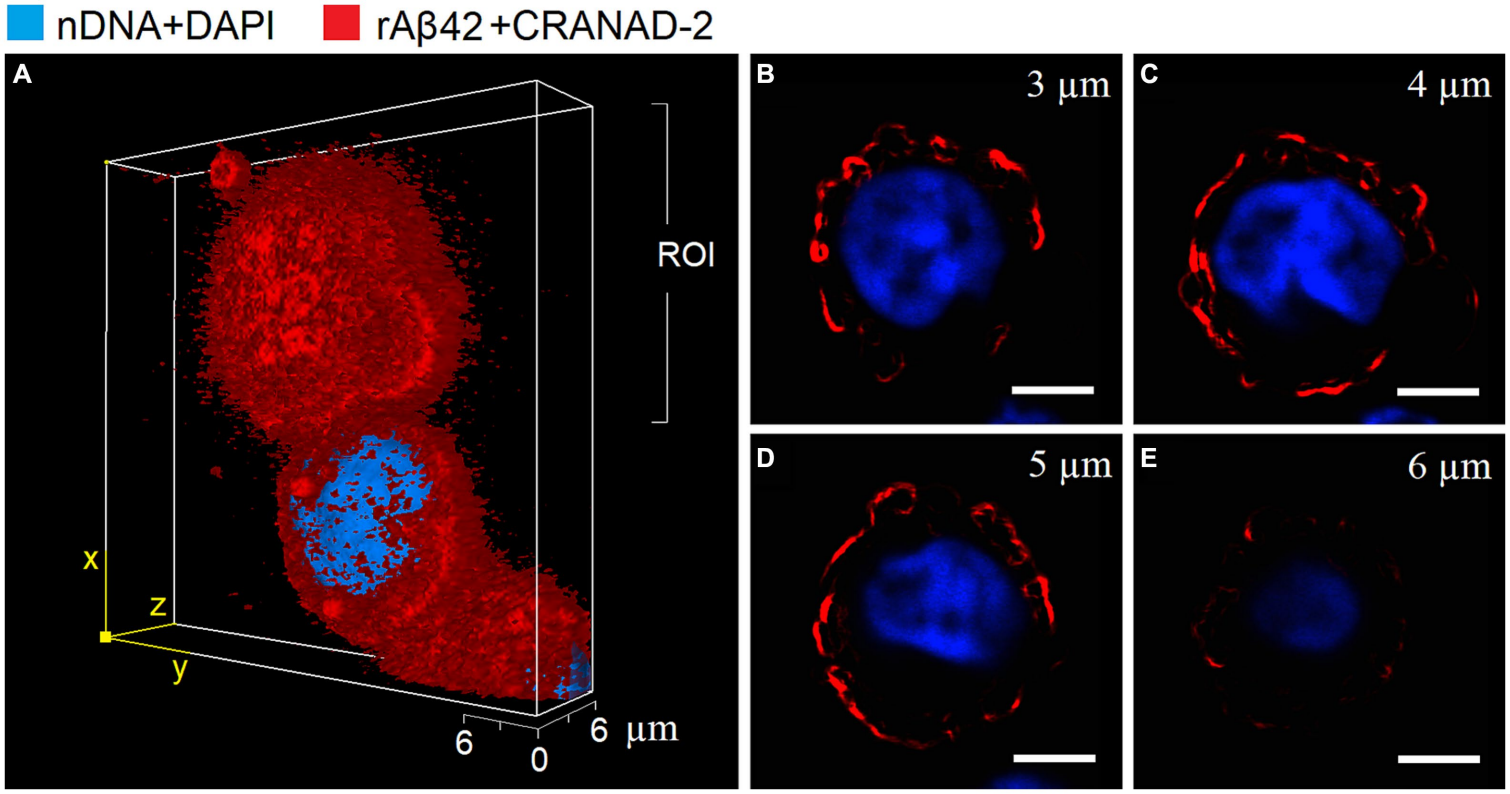
The representative 3D imaging **(A)** of suspended hPBMCs (*n* = 50, 1n = single analyzed cell) after 24 h incubation with rАβ42 (15 nM) by laser scanning confocal microscopy. The dataset is assembled from 4 optical sections in region of interest (ROI, x–z plane) with 1 μm step sizes **(B–E)**. Blue – nuclear DNA staining by DAPI dye; red – rАβ42 staining by CRANAD-2. Scale bar = 5 μm.

By measuring the release of cytokines into supernatant by peripheral blood mononuclear cells in the presence or absence of an exogenous rAβ42 stimulator, it is possible to evaluate the proinflammation activation state of these cells in suspension or in an immobilized state, respectively. Our hypothesis is that the enhancement of PBMCs response is possible in the presence of cell-matrix adhesion. To be able to test this hypothesis, we first assessed cytokine production by free and immobilized PBMCs in the presence or absence of the exogenous rAβ42 stimulator. It was determined that the content of TNFα and Aβ40 in the absence of rAβ42 in the incubation medium does not change after 1 and 24 h of incubation of cells in suspension and in an immobilized state. However, the content of IL1β in both cultures after 24 h of incubation in the rAβ42-free medium was lower compared to 1 h of incubation and its absence. During incubation of cells *in vitro* with exogenous rAβ42 in suspension and in the immobilized state, an increase in the intracellular content of TNFα was observed after 1 and 24 h of incubation and no accumulation of IL-1β in the cells. TNF-α is known to directly suppress the expression of Aβ-degrading enzyme (Miao et al., 2023). In this regard, it is of interest to study the ratio of Aβ42/Aβ40 in cell cultures by immunoenzymatic method and confocal microscopy. It was shown that incubation with rAβ42 led to an increase in the intracellular content of endogenous Aβ40 in cells cultured in suspension and in GEM carriers (Tables 1, 2). The results of determining the content of endogenous Aβ40 by the immunoenzymatic method are also confirmed by visualization with confocal microscopy followed by ratiometric analysis - an increase in the fluorescence intensity of the Aβ40-MCAAD-3 conjugate is observed, which indicates the intensification of the synthesis and accumulation of Aβ40 in the cytoplasm of cells both in suspension and in the composition of GEM carriers after 24 h of incubation with rAβ42. It was shown that rAβ42 fluorescent aggregates were localized on the outer surface of the plasma membrane of cells both in suspension and in alginate carriers (Figures 1A–H, 2A–H). Three-dimensional reconstruction of single cells in suspension (Figure 3A) based on a series of optical sections (Figures 3B–E) also confirms this localization of rAβ42 aggregates. It can be seen that Aβ42 aggregates cover the entire periphery of the cells after 24 h of incubation. These observations are confirmed by the results of other studies, which showed that after 16 h of incubation of PC12 cells with rAβ42, a sharp increase in the accumulation of Aβ42 aggregates was observed at the periphery of the cells, and the maximum accumulation was observed exactly after 24 h of incubation (Kuragano et al., 2020).

**Table 1:** The content of TNFα, IL1β та Аβ40 (ng/g total protein) in suspended hPBMCs without and after incubation with rАβ42 (15 nM).

Parameters	Conditions	Time of incubation, hours		
		0	1	24
TNFα	− rАβ42	21.8 ± 3.5	25.3 ± 2.8	31.5 ± 4.7
	+ rАβ42	26.5 ± 3.8	53.7 ± 4.0*(↑)	108.3 ± 11.5** (↑)
IL1β	− rАβ42	317.4 ± 17.2	331.8 ± 15.3	285.2 ± 15.6*(↓)
	+ rАβ42	302.9 ± 15.7	319.5 ± 17.3	307.6 ± 17.1
Аβ_40_	− rАβ42	33.7 ± 4.1	42.0 ± 3.7	40.3 ± 4.3
	+ rАβ42	38.5 ± 4.5	46.8 ± 4.0	67.5 ± 5.1** (↑)

Data are expressed as means ± SEM (*n* = 24 for each condition; 1*n* = 1 separately incubated cell suspension). According to one-way ANOVA followed by Tukey test for multiple comparisons, the means marked * are significantly different (*p* < 0.05) from previous hour of incubation and the means marked ** are significantly different (*p* < 0.05) from 0 h of incubation and previous hour of incubation; ↓↑ - changes direction.

**Table 2:** The content of TNFα, IL1β та Аβ40 (ng/g total protein) in immobilized hPBMCs without and after incubation with rАβ42 (15 nM).

Parameters	Conditions	Time of incubation, hours		
		0	1	24
TNFα	− rАβ42	31.5 ± 4.2	34.7 ± 4.0	36.0 ± 4.1
	+ rАβ42	37.3 ± 4.5	73.9 ± 5.5** (↑)	161.6 ± 9.2** (↑)
IL1β	− rАβ42	362.7 ± 15.5	370.5 ± 15.1	304.1 ± 15.5*(↓)
	+ rАβ42	377.5 ± 16.2	364.1 ± 15.8	373.6 ± 15.1
Аβ_40_	− rАβ42	39.2 ± 4.5	40.9 ± 4.8	45.7 ± 4.0
	+ rАβ42	43.7 ± 4.0	48.3 ± 4.5	89.2 ± 4.2** (↑)

Data are expressed as means ± SEM (*n* = 24 for each condition; 1*n* = 1 separately incubated immobilized cells culture). According to one-way ANOVA followed by Tukey test for multiple comparisons, the means marked * are significantly different (*p* < 0.05) from previous hour of incubation and the means marked ** are significantly different (*p* < 0.05) from 0 h of incubation and previous hour of incubation; ↓↑ - changes direction.

It is known that the ratio of Aβ42/Aβ40, and not the total level of amyloid peptides, plays a decisive role in the direction and intensity of amyloidogenesis in human neuronal cells (Kwak et al., 2020). Direct observations of fluorescence intensity cannot provide insight into the Aβ42/Aβ40 ratio with binding to peptide localization, as the gradient of their fluorescence intensities in the cell volume is very wide. In this regard, to assess the impact of rAβ42 to amyloidogenesis in PBMCs a ratiometric analysis was performed with the calculation of the index R (rAβ42/Aβ40) – the ratio of rAβ42-specific fluorescence intensity to Aβ40-specific fluorescence intensity.

The ratiometric index R (rАβ42/Аβ40) for suspension cells changes from 0.08 (without incubation with rАβ42) to 0.76 (24 h of incubation with rАβ42), and for immobilized cells from 0.06 (without incubation with rАβ42) to 0.42 (24 h of incubation with rАβ42). That is, under conditions of cell immobilization, the rAβ42/Aβ40 ratio decreases (Figures 1D,H, 2D,H). The ratiometric index R (rАβ42/Аβ40) also gives an idea, firstly, of the degree of cell heterogeneity regarding the presence of both types of amyloid peptides in the plasma membrane and cytoplasm, and, secondly, of the heterogeneity of the intensity of the cell response to an exogenous stimulus. Immobilized cells were significantly less homogeneous in terms of rAβ42/Aβ40 ratio both in the plasma membrane and in the cytoplasm compared to cells in suspension. However, regardless of the type of culture, aggregates of amyloid peptide rАβ42 are localized mainly on the periphery of cells and, to a much lesser extent, in the cytoplasm. This result is consistent with studies demonstrating the ability of human cells to internalize both exogenous monomers and Aβ42 oligomers with their subsequent intracellular aggregation (Nazere et al., 2022).

We found that blocking beta-1 integrins did not affect TNFα and Aβ40 concentrations in hPBMCs after 24 h of cultivation in suspension with exogenous rAβ42. The hPBMCs immobilization after incubation with the β1 integrins blocking antibodies showed a remarkable inhibition of TNFα and Aβ40 accumulation in cells after 24 h of cultivation with exogenous rAβ42 (Table 3) but these parameters were still higher than control values (cells in suspension).

**Table 3:** The content of TNFα and Аβ40 (ng/g total protein) in suspended and immobilized hPBMCs without and after 24 h’ incubation with rАβ42 (15 nM) and blocking β1 integrins.

Parameters	Conditions	Culture formats	
		Suspended cells	Immobilized cells
TNFα	− rАβ42/− blocking β1 integrins	33.7 ± 4.2	38.1 ± 4.5
	+ rАβ42/− blocking β1 integrins	114.6 ± 9.2*	179.5 ± 8.2*
	− rАβ42/+ blocking β1 integrins	35.2 ± 3.8	40.9 ± 4.7
	+ rАβ42/+ blocking β1 integrins	117.2 ± 10.5*	72.8 ± 5.5**
Аβ_40_	− rАβ42/− blocking β1 integrins	45.5 ± 3.8	48.2 ± 4.3
	+ rАβ42/− blocking β1 integrins	71.4 ± 5.5*	93.1 ± 5.8*
	− rАβ42/+ blocking β1 integrins	41.3 ± 3.2	45.6 ± 4.0
	+ rАβ42/+ blocking β1 integrins	73.5 ± 6.1*	77.9 ± 5.5**

Data are expressed as means ± SEM (*n* = 24 for each condition; 1*n* = 1 separately incubated cell suspension or immobilized cells culture, respectively). According to one-way ANOVA followed by Tukey test for multiple comparisons, the means marked * are significantly different (*p* < 0.05) from cells incubated without rАβ42 and without blocked β1 integrins and the means marked ** are significantly different (*p* < 0.05) from cells incubated with rАβ42 and without blocked β1 integrins; ↓↑ - changes direction.

## 4 Discussion

The decrease in the concentration of IL1β in both cultures after 24 h of incubation in the rAβ42-free medium compared to 1 h of incubation and its absence can be explained by the degradation of IL1β by ubiquitin-dependent proteolysis, shown for the spontaneous synthesis of this cytokine in the absence of exogenous stimulation (Sokolik et al., 2016; Behzadi et al., 2022).

Incubation with recombinant Aβ42 leads to its accumulation on the periphery and in the cytoplasm of cells. Penetration of this peptide into the cell is possible by macropinocytosis. In particular, this possibility was shown for astrocytes – the cells internalized both monomeric and oligomeric Aβ42 (Li et al., 2014). In the conditions of short-term incubation with Aβ42, in the process of vesicular transport of Aβ42, the peptide is quickly disposed of after the nutrient medium is freed from it. However, with continuous exposure to extracellular Aβ, accumulation and aggregation of vesicular Aβ occurs due to overload of the endosomal/lysosomal pathway (Hu et al., 2009; Chung et al., 2022). Extracellular Aβ, which is released as a result of proteolytic degradation of APP via the amyloidogenic pathway, forms cytotoxic oligomers by self-aggregation at the cell periphery (Ratan et al., 2023). The presence of Aβ40 is shown in the cells of both cultures even without exogenous stimulation, which is consistent with the results (Haass et al., 1992; Selkoe, 2006) regarding the presence of this peptide in cells as a product of their normal metabolism.

It is known that the ratio of Aβ42/Aβ40, and not the total level of amyloid peptides, plays a decisive role in the direction of amyloidogenesis *in vitro* (Kwak et al., 2020) and the development of Alzheimer’s disease *in vivo* (Kapoor et al., 2023). It was shown that under conditions of cell immobilization, the rAβ42/Aβ40 ratio decreases. This result can be explained by the peculiarities of the cells in the immobilized state compared to the conditions of cell cultivation in suspension. The model of cell cultivation in suspension does not include the factor of cell activation by adhesion. hPBMCs have a number of important adhesion molecules responsible for cell–cell and cell-matrix contacts: selectins, integrins, Ig-like receptors and cadherins. Interactions between cells and the extracellular matrix are critical for processes controlling cell proliferation, activation, migration, and survival (Wennström and Nielsen, 2012). A study (Zhang et al., 2010) showed that decreasing cell density significantly increased levels of Aβ40, Aβ42, total Aβ, and the Aβ42/Aβ40 ratio. It is likely that the immobilization of cells in the alginate hydrogel shields the cells from each other, reducing the degree of cell–cell contact. A decrease in the ratio of Aβ42/40 against the background of an increase in the level of insoluble fibrils and aggregates of Aβ is shown in the cultivation of human neurons in conditions of 3D-culture based on hydrogels (Kwak et al., 2020). This result is explained by the effect of adhesion receptors cadherins and integrins on the release of Aβ (Biose et al., 2023). In addition, the aggregation of Aβ42 on the cell surface increases with an increase in the degree of polymerization of F-actin under conditions of an increase in the number of cell-matrix contacts (Kommaddi et al., 2018). The beta-1 integrins blocking does not affect the concentrations of TNFα and Aβ40 in hPBMCs after 24 h of cultivation in suspension with exogenous rAβ42. Cells in suspension were not affected by the factor of cell activation by cell to matrix adhesion. In hPBMCs, there are many essential adhesion molecules that determine the formation of their subpopulations and functional specificity (Vanoni et al., 2022) such as integrins family. Modification of alginate with an RGD (Arg-Gly-Asp) peptide, an integrin binding ligand, in process of GEM microcarriers formation promoted hPBMCs adhesion by RGD-integrins contacts (Pang et al., 2023). The incubation with the β1 integrins blocking antibodies decrease cells attachment to the alginate matrix. The hPBMCs immobilization after incubation with the β1 integrins blocking antibodies showed a remarkable inhibition of TNFα and Aβ40 accumulation in cells after 24 h of cultivation with exogenous rAβ42 but these parameters were still higher than the parameters in cells in suspension. We suppose that this is due to the increased concentration of TGFα in rAβ42-treated immobilized cells, as TGFs regulates expression integrins (Liu et al., 2022). The increased TGFα level up-regulates *de novo* expression of β1 integrins and promote focal adhesion assemblies for 24 h of cultivation after blocking integrin-mediated cell-matrix interaction.

An important advantage of the technique of immobilized cells is the ability to perform functional studies on the biology of PBMCs, since, unlike transwell-based strategies, it allows good visualization of the cells during the analysis. Overall, this assay enables to interrogate how Aβ42 stimulation may affect PBMCs *in vitro* under static or dynamic immobilization conditions. Monocytes make up a smaller portion of the human PBMC sample than lymphocytes – roughly 10–30 percent. When monocytes are stimulated, they can differentiate into macrophages or dendritic cells. In suspension, monocytes maintain non-adherent state to prevent differentiation. A 3D gel-like microenvironment induces a positive-feedback loop of adhesion activation to facilitate differentiation (Bhattacharya et al., 2018). There is evidence that microglial cells are a key mediator of damage in Alzheimer’s disease that may arise to a greater part from activation and transmigration of monocytes as a result of monocytic cell adhesion molecules are decreased (Hochstrasser et al., 2010) and the cell migration receptors content is increased in Alzheimer’s disease patients (Huang et al., 2023).

Further larger longitudinal studies should then clarify whether any of Aβ42 treated PBMCs adhesion state or adhesion-dependent cells responses may be useful as a diagnostic biomarker for development of novel therapeutic strategies for Alzheimer’s disease.

## 5 Conclusion

The presented experimental results testify that the basic levels of indicators and the intensity of the response of immobilized cells to the exogenous Aβ42 stimulus are significantly greater than those of cells in suspension. Activation of signal transduction and synthesizing activity of mononuclear cells of human peripheral blood is possible (or significantly increases) in the presence of integrins-mediated cell-matrix adhesion. Failure to consider the cell adhesion factor and the use of classical suspension culture may be the cause of false conclusions in the study of exogenous stimuli on hPBMCs obtained from healthy donors and donors with Alzheimer’s disease.

## Data availability statement

The original contributions presented in the study are included in the article/supplementary material, further inquiries can be directed to the corresponding author.

## Ethics statement

Ethical approval was not required for the studies on humans in accordance with the local legislation and institutional requirements because only commercially available established cell lines were used.

## Author contributions

KK: Conceptualization, Data curation, Formal analysis, Investigation, Methodology, Project administration, Resources, Visualization, Writing – original draft, Writing – review & editing. YK: Conceptualization, Data curation, Formal analysis, Funding acquisition, Investigation, Methodology, Resources, Visualization, Writing – original draft, Writing – review & editing. RK: Data curation, Formal analysis, Investigation, Writing – original draft. HA: Methodology, Validation, Writing – review & editing. OT: Methodology, Validation, Writing – review & editing. YB: Funding acquisition, Project administration, Supervision, Writing – review & editing. SS: Conceptualization, Funding acquisition, Project administration, Supervision, Writing – review & editing.
